# Excess healthcare burden during 1918-1920 influenza pandemic in Taiwan: implications for post-pandemic preparedness

**DOI:** 10.1186/1471-2458-11-41

**Published:** 2011-01-17

**Authors:** Ying-Hen Hsieh, Chi-Ho Chan

**Affiliations:** 1 Department of Public Health and Center for Infectious Disease Epidemiology Research, China Medical University, Taichung, Taiwan; 2 Department of Microbiology and Immunology, Chung Shan Medical University, Taichung, Taiwan

## Abstract

**Background:**

It is speculated that the 2009 pandemic H1N1 influenza virus might fall into a seasonal pattern during the current post-pandemic period with more severe clinical presentation for high-risk groups identified during the 2009 pandemic. Hence the extent of likely excess healthcare needs during this period must be fully considered. We will make use of the historical healthcare record in Taiwan during and after the 1918 influenza pandemic to ascertain the scope of potential excess healthcare burden during the post-pandemic period.

**Methods:**

To establish the healthcare needs after the initial wave in 1918, the yearly healthcare records (hospitalizations, outpatients, etc.) in Taiwan during 1918-1920 are compared with the corresponding data from the adjacent "baseline" years of 1916, 1917, 1921, and 1922 to estimate the excess healthcare burden during the initial outbreak in 1918 and in the years immediately after.

**Results:**

In 1918 the number of public hospital outpatients exceeded the yearly average of the baseline years by 20.11% (95% CI: 16.43, 25.90), and the number of hospitalizations exceeded the corresponding yearly average of the baseline years by 12.20% (10.59, 14.38), while the excess number of patients treated by the public medics was statistically significant at 32.21% (28.48, 39.82) more than the yearly average of the baseline years. For 1920, only the excess number of hospitalizations was statistically significant at 19.83% (95% CI: 17.21, 23.38) more than the yearly average of the baseline years.

**Conclusions:**

Considerable extra burden with significant loss of lives was reported in 1918 by both the public medics system and the public hospitals. In comparison, only a substantial number of excess hospitalizations in the public hospitals was reported in 1920, indicating that the population was relatively unprepared for the first wave in 1918 and did not fully utilize the public hospitals. Moreover, comparatively low mortality was reported by the public hospitals and the public medics during the second wave in 1920 even though significantly more patients were hospitalized, suggesting that there had been substantially less fatal illnesses among the hospitalized patients during the second wave. Our results provide viable parameters for assessing healthcare needs for post-pandemic preparedness.

## Background

The 2009 pandemic H1N1 (pH1N1) virus spread swiftly to all parts of the world in a matter of a few months after it was first identified in Mexico in March. In August 2010, World Health Organization (WHO) declared the world to be in the post-pandemic period, when the pH1N1 virus is expected to continue to circulate as a seasonal virus for some years to come [1]. Moreover, active transmission of pandemic influenza virus still persists in some local areas, and it is still unclear whether the pandemic influenza activity has already transitioned into a seasonal pattern [2]. It is further speculated that groups identified during the recent pandemic as at a higher risk of severe or fatal illness will probably remain at a heightened risk during the post-pandemic period, although the number of such cases may decrease. In addition, a small proportion of people infected during the 2009 pandemic developed a severe form of primary viral pneumonia that is not commonly seen during seasonal epidemics and is especially difficult to treat. Therefore, quantitative ascertainment of the likely healthcare burden is an important aspect of post-pandemic preparedness planning.

More than 90 years ago, the first pandemic of the last century was initially observed in the early spring of 1918. It was quickly followed by much more fatal second and third waves in the fall and winter of 1918-1920, causing an estimated 50 million deaths [3]. It still proves to be a major dilemma for the scientific community to understand what had happened precisely, how it had happened, and why it was in several ways unlike any other influenza pandemic in recorded human history [3]. Several studies have focused on quantifying the global impact of that pandemic, either by using records of cases and mortality of the affected countries (e.g., [4-6]), or by using vital statistics data from the affected countries to estimate the excess mortality of these countries. For example, Murray et al. [6] estimated the excess mortality rate of each affected country and extrapolated to conclude that an estimated 62 million people would be killed in a similar pandemic in 2004. However, to the best of our knowledge historical healthcare records had not been used to directly assess the excess healthcare burden during a pandemic.

The 1918-1920 pandemic swept through Taiwan in two distinct waves, both occurred during winter influenza seasons - the first at the end of 1918 and the second in early 1920, and with devastating loss of human lives. Increased influenza cases were initially reported in mid-October 1918 in Keelung, the main seaport in north [7]. A report published in the Taiwan Medical Association Journal in February 1920 [8] on the devastation brought by this first wave of influenza outbreak reported that 20.8% of the population had been infected with a case fatality rate of 3.26%. A second wave appeared at the end of 1919 and affected Taiwan through the early months of 1920, also with a severe death toll. A recent study [9] made use of the monthly mortality data in Taiwan during that time period to estimate that the total number of excess deaths during the pandemic months of November-December 1918 and January-February 1920 was 51,048 (95% CI 41,998-61,853).

The 1918-1920 influenza epidemic in Taiwan was intriguing in several aspects. First, it was one of the few regions in the world that a wave had occurred as late as 1920 [5]. Moreover, the two waves of the epidemic were separated by almost a full year, in contrast to intervals of a few months in most countries in the world [3], and both occurred during the months of yearly winter influenza season in Taiwan. The relatively late occurrence of the initial outbreak in November of 1918, as well as the second wave in early 1920, perhaps signifies the relative lack of international travel due to its status at that time as a fairly recent Japanese colony (since 1895), in contrast to nearby regions such as Singapore which is geographically similar but more globally connected [10].

Moreover, Taiwan is located in the tropical-subtropical zone with similar excess influenza deaths to those observed in temperate zone during periods of previously recognized influenza epidemics in Taiwan [11,12], and has been known to be one of the evolutionarily leading regions for global circulation of influenza [13]. Therefore, the island population in Taiwan could serve as a good model for studying spatial and temporal spread of influenza outbreak in a confined region during distinct waves of a pandemic. In this study, we will make use of the healthcare records from 1918-1920 in Taiwan to examine the level of excess healthcare burden under which healthcare system was extended in the years immediately following the initial wave of the pandemic in 1918, and to ascertain the possible post-pandemic demands on a modern healthcare system, such as we might face in the coming influenza seasons.

## Methods

Our main source of data is the 1895-1945 Statistical Abstract of Taiwan [14] which contains the complete and detailed vital statistics of Taiwan during all 50 years of the Japanese occupation including detailed yearly healthcare records. We will use this data to explore the public health events that had occurred during those years during and immediately after the initial epidemic in 1918. During 1918-1920, there were 12 large public hospitals, 18-19 smaller public hospitals, and 60-68 private hospitals in Taiwan. In addition, there was a large network of trained "public medics" which was responsible for, among other duties, providing basic and primary medical care in the local community for people with clinical symptoms and for reporting local incidence of illnesses (including epidemic intelligence) to the government [15]. However, only the numbers of outpatients, in-patient hospitalizations, and all-cause deaths for the large public hospital and the public medics system were given in the Statistics Abstract [14].

A measure of the severity of an epidemic and its burden on the healthcare system is the excess number of hospital visits and hospitalized patients during the epidemic and during the post-epidemic years. Yearly excess numbers of outpatients, in-patient hospitalizations (abbreviated to "hospitalization" hereafter), and all-cause deaths reported by the large public hospital and the public medics system during 1918-1920 were computed by the method of Serfling et al. [16]. We first computed the yearly mean numbers of outpatients, hospitalizations, and all-cause deaths over the two adjacent baseline years before the epidemic (1916, 1917) and the two adjacent baseline years after (1921, 1922). We then subtracted these means from the corresponding yearly numbers of outpatients, hospitalizations, and all-cause deaths for each year during 1918-1920 to obtain the yearly excess numbers during 1918-1920. A yearly excess number is considered to be statistically significant if the number (of outpatients, hospitalizations, or all-cause deaths) for that pandemic year exceeds the corresponding mean of the adjacent baseline years of 1916, 1917, 1921, and 1922 by 2 SDs or more [9]. In order to compare the yearly excess healthcare burden of the pandemic years of 1918-1920, we computed the percentages of these yearly excess numbers over the means of the adjacent baseline years, to ascertain the impact of the pandemic on the healthcare system during each of the years in 1918-1920.

## Results

**Figure 1:**
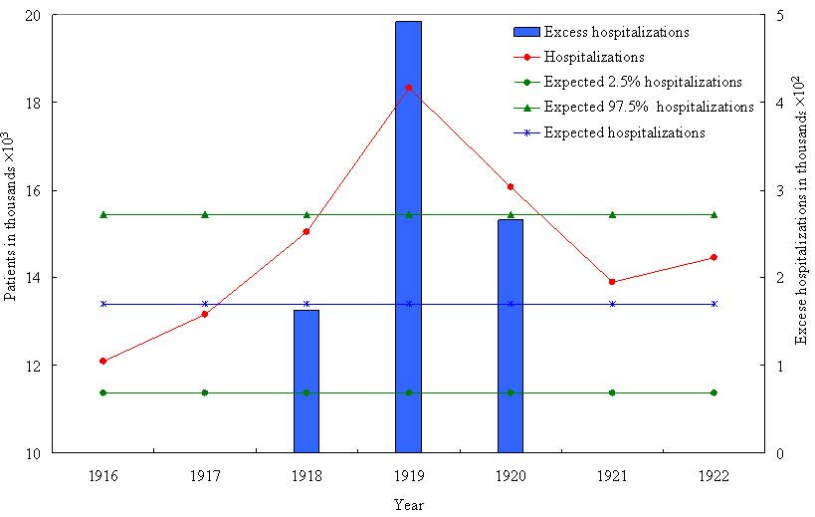
**The yearly excess number of hospitalized patients reported by 12 public hospitals during 1918-1920 compared with the yearly averages during the adjacent "baseline" years of 1916, 1917, 1921, and 1922**. Blue bars denoting the excess numbers are scaled to the right side of the figure.

**Figure 2:**
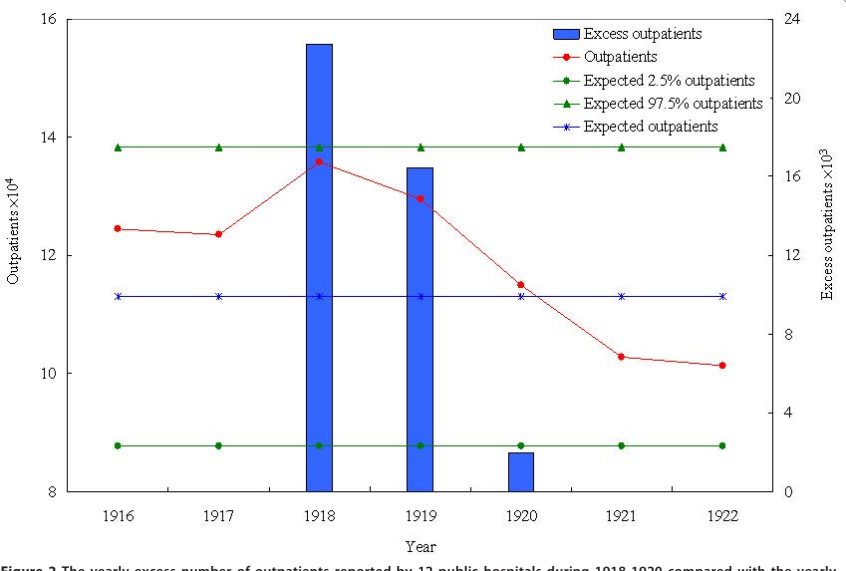
**The yearly excess number of outpatients reported by 12 public hospitals during 1918-1920 compared with the yearly averages during the adjacent "baseline" years of 1916, 1917, 1921, and 1922**. Blue bars denoting the excess numbers are scaled to the right side of the figure.

**Figure 3:**
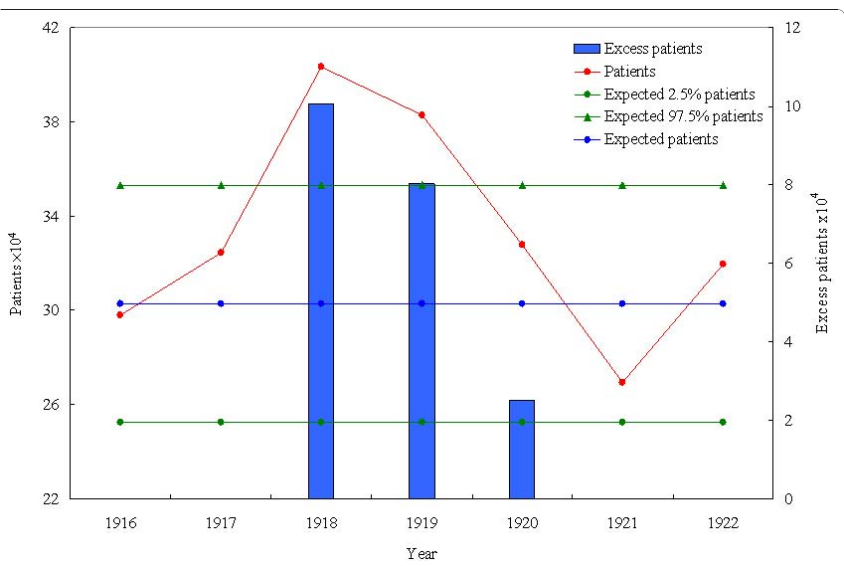
**The yearly excess number of patients reported by public medics system during 1918-1920 compared with the yearly averages during the adjacent "baseline" years of 1916, 1917, 1921, and 1922**. Blue bars denoting the excess numbers are scaled to the right side of the figure.

The yearly excess number of patients and all-cause deaths reported by 12 public hospitals and public medics system during 1918-1920 compared with the yearly averages during the adjacent "baseline" years of 1916, 1917, 1921, and 1922 are shown in Figures 1, 2, 3. The percentages of the excess number of medical treatments and hospitalization for each year during 1918-1920 over the averaged yearly numbers of the adjacent baseline years of 1916, 1917, 1921, and 1922 are given in Table 1 with the 95% confidence intervals (CI). In 1919, the numbers of hospitalizations and treatments by public medics are clearly excessive, exceeding even the corresponding numbers in the epidemic years in 1918 and 1920 in some instances (see Figures 1, 2, 3). In 1918 there is a significant increase in treatments by the public medics (32.21%; 95% CI: 28.48, 39.82); however, the increases in public hospital outpatients (20.11%; 95% CI: 16.43, 25.90) and hospitalized patients (12.20%; 95% CI: 10.59, 14.38) were not statistically significant. In 1920, only the increase in hospitalizations was statistically significant (19.83%; 95% CI: 17.21, 23.38).

**Table 1:** Percentage of the excess numbers of medical treatments and hospitalization as reported by 12 large public hospitals and public medics for each year during 1918-1920 over the averaged yearly numbers of the adjacent baseline years of 1916, 1917, 1921, and 1922.

Year	Public Hospitals		Public medic patients
		
	Outpatients (95% CI)	# of hospitalizations	
1918	20.11 (16.43, 25.90)	12.20 (10.59, 14.38)	32.21* (28.48, 39.82)
1919	14.55 (11.89, 18.74)	36.70* (31.85, 43.27)	26.64* (22.70, 31.73)
1920	1.72 (1.40, 2.21)	19.83* (17.21, 23.38)	8.27 (7.09, 9.92)

*Statistically significant (more than 2 SD).

Moreover, the percentages of excess yearly number of deaths reported by 12 large public hospitals and public medics for each year during 1918-1920 over the averaged yearly number of deaths of the adjacent years of 1916, 1917, 1921, and 1922, are given in Table 2. Only the excess numbers of deaths in 1918 are significant, at 29.90% (24.80, 37.62) for the number of deaths reported by the 12 large public hospitals and 23.90 (20.44, 28.77) by the public medics.

**Table 2:** Percentage of the excess number of deaths reported by 12 large public hospitals and public medics for each year during 1918-1920 over the averaged yearly number of deaths of the adjacent baseline years of 1916, 1917, 1921, and1922.

% Excess Mortality reported	Public hospitals	Public medic system
Excess in 1918*	29.90 (24.80, 37.62)	23.90 (20.44, 28.77)
Excess in 1919	11.93 (9.90, 15.02)	-3.12 (-2.67, -3.76)
Excess in 1920	16.49 (13.70, 20.78)	9.24 (7.90, 11.12)

*Excess deaths in 1918 are statistically significant (more than 2 SD).

## Discussion

There is an underlying assumption of our method that there is no drastic change in the Taiwanese population during 1916-1922, when the population size increased steadily but only by less than 10%, from 3,596,109 to 3,904,692 [14]. Methods to detect significant changes over time can be found in, among others, [17].

We also note that the decline following 1919 in all the three sets of numbers reflecting healthcare burden might be partly attributable to a regression to the mean, given that the second wave was still in full force in the first two months of 1920. The limitation in the data, where only yearly numbers (and not monthly numbers) are given, makes it impossible to determine the months in which the drop had occurred, and whether the decline is attributable to the decrease in healthcare demand *after *the pandemic was over or to the low level of healthcare demand even during the pandemic months early in the year.

The percentages of excess deaths reported by the public hospitals and the public medics in 1918 were both statistically significant, corroborating the results from another study [9]. Moreover, given that the excess hospitalizations in the public hospital were not statistically significant and yet the percentage of excess deaths reported by the public hospitals was statistically significant and exceeded even those reported by public medics, one could infer that a comparatively larger proportion of hospitalized patients had lost their lives in 1918. However, the corresponding percentages of excess deaths were not statistically significant in 1920, even though similar levels of excess numbers of deaths were found in both waves [9]. This gives indication that the second wave in early 1920, although with a significantly greater number of hospitalizations, had substantially fewer fatal illnesses among the hospitalized patients when compared with the initial wave in 1918.

## Conclusions

Our results indicate that there was a considerable extra burden on the public medic system during the initial wave of the epidemic in 1918, with a significant loss of lives reported by both the public medic system and the 12 large public hospitals. In comparison, only a substantial number of excess hospitalizations in the public hospitals was reported in 1920, indicating that the population was relatively unprepared for the first wave in 1918 and did not fully utilize the public hospital system.

The most surprising part of our findings is the significant increases in the numbers of hospitalizations and treatments by the public medics for 1919, the year between the two waves when only the beginning of the second wave in December of 1919 had contributed some initial influenza deaths over the monthly means of neighboring "baseline" years [9]. One possible reason for this is the contribution to hospitalization/treatment due to other diseases that were prevalent in 1919 (e.g., a cholera outbreak which led to 2,693 deaths in 1919 and 1,675 deaths in 1920 [14]). However, limited by the retrospective nature of the study design, we are unable to identify or rule out other non-relevant diseases or conditions solely from our hospitalization/treatment data due to the lack of more detailed historical data.

It is also possible that the severe first epidemic wave during the previous winter of 1918 had alarmed the population to being more readily willing to quickly seek medical assistance at the first sign of an ailment, even though many of these illnesses might be unrelated to influenza. That is, the populace was more readily alerted to seek treatment from local public medics with any initial symptom of illness (as compared to visiting large hospital), while patients with more severe illness (of any kind) are more likely to be hospitalized by physicians. This type of overreaction on the part of the healthcare system and the general public had also been observed during the 2003 SARS outbreak where many non-SARS patients were hospitalized unnecessarily as suspected SARS cases. Adding the fact that both the numbers of hospitalizations and treatments by the public medics dropped drastically next year in 1920, the last scenario seems plausible. Another possibility is that pathophysiological or social processes [18] may be at play where the end of World War I could have contributed to movement of people and affected the pandemic's spread, although the Taiwan data indicated no noticeable increase in migration,.

Our results suggest that the excess burden on the healthcare system was high in the post-pandemic period, which would be a major challenge to any well-managed healthcare system. But it could contribute to fewer fatal illnesses. It has been noted that any present-day projection based on the 1918-1920 pandemic merely presents a worst-case scenario which we can avoid with diligence [19,20]. However, one should note that the situation today, 90 years later, is very different in many aspects. While modern communication systems may facilitate more rapid spread of infections, implementation of interventions (school closures, masks, hand washing, bans on spitting in public, etc.) may reduce the overall transmission of influenza. Moreover, population demographics, health status and prior exposure to influenza are also different. In 1918-1920 life expectancy was shorter, so the population would have been on the average younger with less prior exposure to influenza, and therefore less compounded by past circulation of influenza as mentioned previously. Our results provide a basis to learn from the past to obtain projections of pandemic scenario and the viable hypothetical parameters for assessing healthcare needs specifically for the current post-pandemic preparedness in every country, including antivirals and vaccines needs for speedy, adequate, and equitable distribution. Finally, while this study is retrospective in design, the study methods can be easily modified for a prospective design and incorporated into a part of syndromic surveillance during a future influenza pandemic to monitor and adjust resources accordingly.

## Competing interests

The authors declare that they have no competing interests.

## Authors' contributions

YHH conceived and organized the study, carried out the analysis, and wrote the first draft of the manuscript. CHC participated in the conception of the study, the collection of the data, and the writing. Both authors read and approved the final manuscript.

## Pre-publication history

The pre-publication history for this paper can be accessed here:

http://www.biomedcentral.com/1471-2458/11/41/prepub
